# To biopsy or not biopsy, that is the question - PI-RADS 3 prostate lesions – validation of clinical and radiological parameters for biopsy decision-making

**DOI:** 10.1186/s12894-025-01986-2

**Published:** 2025-11-01

**Authors:** Toni Franz, Tom Sicker, Julian Lueke, Benny Dinh, Thi Phuc Ho, Theodoros Spinos, Lars-Christian Horn, Alexander Schaudinn, Evangelos Liatsikos, Jens-Uwe Stolzenburg

**Affiliations:** 1 Department of Urology, University of Leipzig, Liebigstraße 20a, Leipzig, 04103 Germany; 2 Department of Urology, University of Patras, Rio Patras, 26500 Greece; 3 Institute of Pathology, University of Leipzig, Liebigstraße 26, Leipzig, 04103 Germany; 4 Department of Diagnostic and Interventional Radiology, University of Leipzig, Liebigstraße 20, Leipzig, 04103 Germany

**Keywords:** PI-RADS 3, Prostate cancer, Detection rate, Fusion biopsy

## Abstract

**Objectives:**

Multiparametric MRI (mpMRI) enhances prostate cancer (PCa) detection, especially when combined with targeted or MRI–ultrasound fusion biopsy. However, PI-RADS 3 lesions remain diagnostically indeterminate, with variable malignancy risk and heterogeneous clinical management. This study aims to identify clinical and radiological predictors of PCa and clinically significant PCa (csPCa) in patients with PI-RADS 3 lesions in order to enhance risk stratification. By disentangling patient- and disease-specific characteristics from imaging findings, the study evaluates their independent prognostic value. The primary objective is to validate non-imaging parameters as reliable tools for risk stratification in indeterminate cases, thereby supporting clinical decision-making when radiological assessment alone is inconclusive.

**Patients and methods:**

In this retrospective cohort study, 671 patients with 981 PI-RADS 3 lesions underwent mpMRI and MRI–ultrasound fusion-guided transrectal biopsy, including both targeted and systematic cores. Histopathological evaluation was based on ISUP grading. Logistic regression models were used to assess associations between clinical/radiological factors and biopsy outcomes.

**Results:**

Overall cancer detection per lesion was 36.9%, with csPCa detected in 15.8% of lesions and 42.8% of positive biopsies. PSA density emerged as the strongest predictor of both PCa and csPCa, while prostate volume was inversely associated. csPCa was more commonly found in patients undergoing primary biopsy and those with posterior lesion localization. In selected low-risk groups, csPCa detection was rare, suggesting potential to avoid unnecessary biopsies, with specificity reaching up to 90%.

**Conclusions:**

Overlapping benign conditions and interobserver variability contribute to uncertainty in the interpretation of PI-RADS 3 lesions with regard to the indication for biopsy. PSA density and clinical context support risk-adapted decision-making, aligning with current guideline recommendations. A personalized approach is recommended to balance the risks of under- and overdiagnosis in managing PI-RADS 3 lesions.

**Supplementary Information:**

The online version contains supplementary material available at 10.1186/s12894-025-01986-2.

## Introduction

Prostate biopsy remains essential for diagnosing prostate cancer and guiding treatment decisions [1, 2]. Despite technological advances, no imaging modality has supplanted histopathological confirmation. However, systematic biopsy alone may miss up to 40% of clinically significant prostate cancers (csPCa) and often leads to overdiagnosis by detecting indolent tumors [3]. The advent of multiparametric MRI (mpMRI) has significantly improved detection rates, particularly when combined with targeted biopsy techniques [4–6].

In current clinical practice, mpMRI plays a key role in prostate cancer diagnosis and aligns with established quality standards [1, 2]. When assessed via the Prostate Imaging Reporting and Data System (PI-RADS), mpMRI enables lesion localization and risk stratification [7]. MRI-ultrasound fusion biopsy further enhances diagnostic precision by targeting mpMRI-identified lesions [8–10]. In biopsy-naïve men, combining systematic and mpMRI-targeted biopsies increases csPCa detection by roughly 10% compared to systematic biopsy alone [10–12]. Nonetheless, both mpMRI and targeted biopsy may still fail to detect certain significant tumors [3, 12].

PI-RADS 3 lesions present a diagnostic challenge. Representing about 17% of prostate MRI reports, they carry a 10–30% probability of harboring csPCa (Gleason ≥ 3 + 4) [13–19]. While most such lesions are benign or indolent, a clinically relevant subset is not. This leads to uncertainty regarding the necessity of performing a biopsy. Consequently, management strategies vary: some centers opt for routine biopsy, while others prefer surveillance, factoring in PSA levels, PSA density, and family history [19, 20].

Further, the variability in radiological interpretation complicates decision-making. The classification of PI-RADS 3 is influenced by image quality and radiologist expertise, leading to interobserver variability [21]. These lesions often lack definitive imaging features of malignancy, prompting delayed intervention through repeat MRI at 6–12 months [20, 21]. This delay may increase patient anxiety and postpone diagnosis.

Thus, PI-RADS 3 lesions represent the “gray zone” in prostate mpMRI, as it does not provide definitive guidance on whether immediate biopsy or surveillance is the optimal approach. The European Association of Urology (EAU) guidelines recommend a patient-specific approach, incorporating PSA density into clinical decision-making [1, 22].

This study investigates the detection rates and predictive factors for prostate cancer and csPCa in patients with PI-RADS 3 lesions on mpMRI. It evaluates both clinical and radiologic predictors of csPCa in mpMRI-targeted biopsy and explores risk stratification strategies based on combined clinical-imaging parameters.

## Patients and methods

A retrospective cohort study was performed involving 671 consecutive patients presenting with a total of 981 lesions classified as PI-RADS 3. All subjects exhibited elevated serum prostate-specific antigen (PSA) levels and normal findings on digital rectal examination (DRE). Inclusion and exclusion criteria are provided in Supplementary Appendix Table S1. Between August 2014 and September 2024, each participant underwent a transrectal magnetic resonance imaging (MRI)–ultrasound fusion-guided biopsy within our clinical institution. Prior to inclusion, written informed consent was obtained from all patients. The diagnostic pathway encompassed a combination of DRE, serum PSA quantification, and mpMRI assessment, followed by targeted biopsy utilizing MRI-ultrasound fusion technology.

In accordance with the legal framework for data processing defined by the local law (*Sächsisches Krankenhausgesetz* § 29), physicians are authorized to process patient data stored within their departments, university hospitals, or affiliated medical institutions for scientific research purposes without obtaining individual informed consent.

The institutional review board of the University of Leipzig approved this study with the ethics.

agreement (reference number 280/25-ek), approved initially on 19/08/2025.

### Multiparametric MRI

Multiparametric MRI was performed using three different 3-Tesla scanners (Magnetom Trio and Magnetom Prisma Fit, Siemens Healthcare, Erlangen, Germany; Ingenia 3.0T, Philips Healthcare, Best, The Netherlands). Imaging in all patients was acquired with a combination of phased-array pelvic and spine coils. Although the protocol underwent minor adjustments during the study period, all examinations consistently adhered to PI-RADS criteria. The standardized mpMRI protocol included T2-weighted imaging (T2w), diffusion-weighted imaging (DWI), and dynamic contrast-enhanced imaging (DCE) following intravenous administration of 15–20 mL of contrast agent (Gadovist, Bayer Healthcare, Berlin, Germany; or Clariscan, GE Healthcare Buchler, Braunschweig, Germany). To reduce bowel peristalsis, all patients received an intravenous injection of either 40 mg butylscopolamine (Buscopan, Boehringer Ingelheim, Ingelheim, Germany) or 1 mg glucagon (Glucagen, Novo Nordisk, Gentofte, Denmark). For the protocol, see Table 1.

**Table 1:** Imaging parameters of multiparametric prostate MRI

	Sequence	Slices	ST [mm]	IPR [mm]	TR [ms]	TE [ms]	FOV [mm]	FA [°]
T2w (tra, cor, sag)	TSE	25–26	3.0.	0.4–0.7	4490	101–108	220 × 220	120
DWI (tra)	EPI	20	3.0	1.6 × 1.6	3000	58	220 × 220	90
T1w DCE (tra)	GRASP	24	3.0	1.1 × 1.1	4.0	1.86	240 × 240	12

*DWI *Diffusion weighted imaging, *DCE *Dynamic contrast enhanced, *TSE *Turbo spin echo, *SS-EPI *Single-shot echo planar imaging, *SPGR *Spoiled gradient echo, *ST *Slice thickness, *IPR *In-plane resolution, *TR *Repetition time, *TE *Echo time, *FOV *Field of view, *FA *Flip angle

**Fig. 1:**
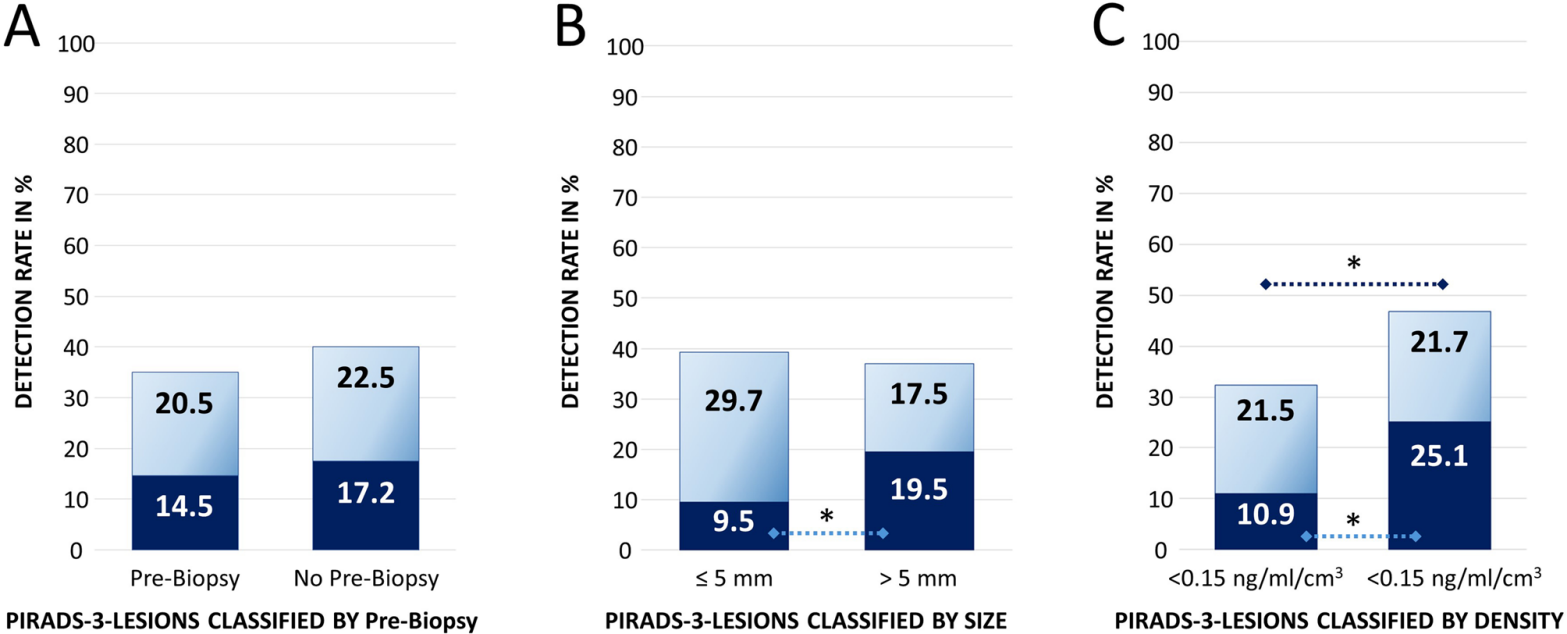
Prostate Cancer Detection Rate and different clinical variables; classification into clinically significant (cs – dark blue) and non-significant (non-cs – light blue) cancers; (**A**) Pre-Biopsy (**B**) Size of the PIRADS-3-Lesion (**C**) PSA-Density; dotted black and dark blue line indicates statistical significance, * *p* < 0.01

All scans were interpreted by board-certified radiologists with over 10 years of experience in prostate imaging all holding the certification of the National Radiological Society for prostate MRI diagnostics, following current standards and reported using PI-RADS Version 2.1. The protocol included T2-weighted, diffusion-weighted (DWI), and dynamic contrast-enhanced (DCE) sequences. Lesion assessment followed zone-specific criteria based on the dominant sequence as defined by the applicable PI-RADS version. Lesions were annotated on T2-weighted images using the institutional PACS and assigned to one of the 10 prostate segments defined in PI-RADS v2.1 [15] (Fig. 1). In addition, lesions were categorized as anterior or posterior and assigned to base, midgland, or apex. Lesion and prostate diameters (in mm) were measured in three dimensions (axial and sagittal T2w), and respective volumes were calculated using the ellipsoid formula (0.5236 × height × width × depth). A novel metric, sagittal lesion depth, was defined as the distance between the posterior border of the index lesion and the posterior prostate surface in sagittal T2w images. For lesions located in the peripheral zone (PZ), DWI served as the primary sequence, while in the transition zone, T2-weighted imaging is the dominant sequence. DWI scores ranging from 1 to 5 were used to estimate the probability of clinically significant prostate cancer. In lesions with an intermediate score (DWI score 3), the DCE sequence was evaluated. Focal early contrast enhancement in DCE prompted upgrading to PI-RADS 4. These patients were excluded from the study. Prior to biopsy, all patients underwent mpMRI, with lesions classified as PI-RADS 3. Biopsy decisions were based on PSA levels, life expectancy, comorbidities, and validated clinical risk tools.

### Fusion biopsy

Prostate tissue sampling was performed transrectally under local anesthesia in an outpatient setting. Patient positioning (lithotomy or left lateral) varied by surgeon preference and spatial constraints. Biopsies were guided by the Koelis^®^ navigation system (Koelis, Grenoble, France). A spring-loaded biopsy gun with an 18-gauge, 200-mm needle was used. Two to three targeted cores were obtained from mpMRI-suspicious lesions, followed by a standard 12-core systematic sampling within the same session. All cores were individually evaluated histologically by a consultant uropathologist. In malignant cases, diagnosis and grading were independently verified by a second board-certified pathologist. Histopathological assessment followed ISUP Gleason grading. Clinically significant prostate cancer was defined as ISUP grade group ≥ 2 [23].

### Measurements and statistical analysis

Patients with prior definitive prostate treatments—such as external beam radiation, brachytherapy, cryoablation, HIFU, or MRI-guided focal laser ablation—were excluded. Since a positive digital rectal examination (DRE) is highly suggestive of clinically significant prostate cancer (csPCa), these patients were excluded from the study.

Cases lacking documentation of PI-RADS score, serum PSA, or prostate volume were also omitted. The dataset, including clinical, imaging, and histopathological variables, underwent descriptive statistical analysis. Data processing was performed using Microsoft Excel and DATAtab (Seiersberg, Austria). Continuous variables were reported as means and interquartile ranges (IQR), categorical data as counts and percentages. Two-sided hypothesis testing (*p* < 0.01 or *p* < 0.05) assessed statistical significance. To control for confounders, univariate and multivariate logistic regressions were conducted. Detection outcomes were evaluated on a per-lesion basis to reflect multiple lesions per patient.

## Results

From August 2014 to September 2024, we identified 709 patients with normal findings on digital rectal examination (DRE) who underwent MRI-ultrasound fusion-guided prostate biopsies at our institution. To ensure analytical precision, 38 patients were excluded: 10 due to prior definitive prostate treatments (e.g., radiation or focal therapy), and 28 due to incomplete documentation.

Consequently, a final cohort of 671 patients with 981 PI-RADS 3 lesions was eligible for thorough inclusion and analysis. Median patient age was 65.44 years, consistent with the target population for prostate cancer screening. The median PSA level was 8.95 ng/ml, and median prostate volume was 61.2 mL.

Patient and lesion characteristics, along with detection rates, are summarized in Table 2.

**Table 2:** Characteristics of patients and targets

**Characteristics**	**All 671 patients**, **981 lesions**	**Interquartile Range (IQR)**
Age in years, mean	65.44	(54.9, 76.0)
PSA level in ng/mL, median	8.95	(4.3, 13.6)
PSA densitiy in ng/mL/cm^3^, median	0.16	(0.06, 0.26)
< 0.15	423 (63.05%)	
≥ 0.15	248 (36.95%)	
Prostate volume in cm^3^, median	61.2	(27.3, 95.1)
Biopsy-naïve	351/671 (52.3%)	
Previous biopsies, mean, min, max	0.80 (0–7)	0–1.05.05
Lesion size in mm, median	10.04	7.1–12.99.1.99
≤ 5 mm	321 (32.72%)	
> 5 mm	660 (67.28)	
Localization		
anterior fibromuscular stroma	27 (2.75%)	
anterior	539 (54.94%)	
posterior	415 (42.3%)	
Diagnosis of all PCa in % per lesion	36.9% (362/981)	
Diagnosis of csPCa in % per lesion	15.8% (155/981)	

Notably, 351 patients (52.6%) had no prior prostate biopsy. The remaining 320 patients (47.4%) had undergone at least one transrectal ultrasound (TRUS)-guided biopsy prior to the MRI-US fusion biopsy, with a mean of 0.80 procedures (range: 0–7). A table with the number of prior biopsies is provided in the Supplementary Appendix as table S2. Older patients exhibited significantly larger prostate volumes (*p* < 0.01), higher PSA levels (*p* < 0.01), and were more frequently referred for biopsy based on elevated PSA rather than abnormal digital rectal examination findings (*p* < 0.01).

### Overall detection

**Fig. 2:**
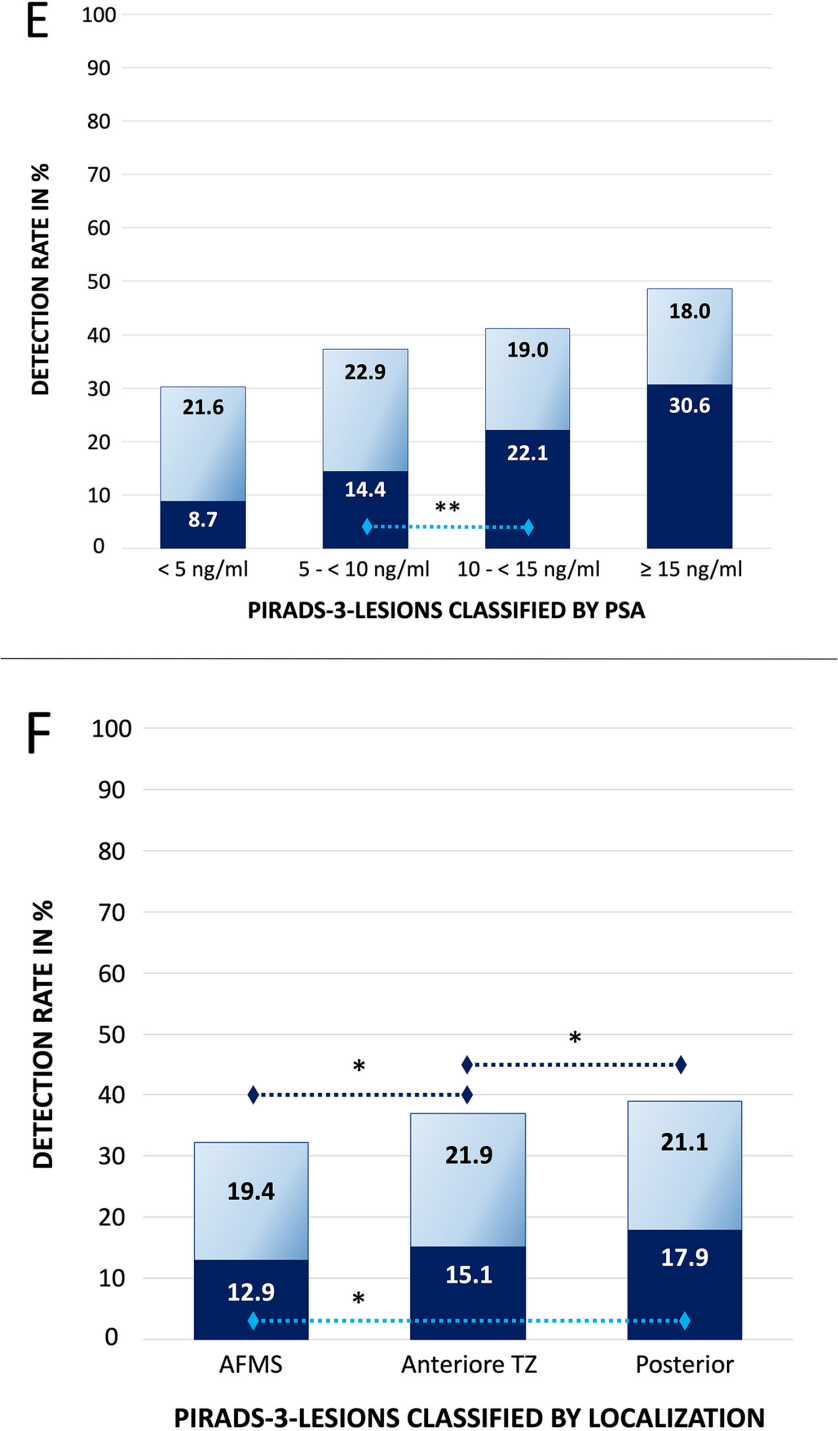
Prostate Cancer Detection Rate and different clinical variables; classification into clinically significant (cs – dark blue) and non-significant (non-cs – light blue) cancers; (**E**) serum PSA (**F**) localization; dotted black and blue line indicates statistical significance, * *p* < 0.01; ** *p* < 0.05

The overall detection rate per target, was 36.9% (362/981 targets). The rate of clinically significant cancers was 15.8% in correlation with the entire cohort (155 csPCa/981 all targets), and 42.8% in correlation to all detected cancers (155 csPCa/362 all cancers). When analyzing the detection rates of all prostate carcinomas and clinically significant prostate carcinomas in relation to potentially influencing variables, notable differences emerge, suggesting variable-dependent diagnostic sensitivity (Table 1; Figs. 1 and 2, supplementary appendix figure S1).

### Univariate und multivariate analysis

To evaluate the diagnostic accuracy of clinical variables in predicting all prostate cancer (PCa) and clinically significant prostate cancer (csPCa) among PI-RADS 3 patients, we conducted logistic regression analyses using both univariate and multivariate approaches.

Univariate analysis identified several variables significantly associated with the presence of all PCa: older age (*p* = 0.003), higher serum PSA levels (*p* < 0.001), greater lesion diameter (*p* = 0.045), and smaller prostate volume (*p* < 0.001). PSA density emerged as the strongest predictor for both endpoints, demonstrating a highly significant association (*p* < 0.001) and the highest area under the curve (AUC) among all single-variable models. The mean lesion size was slightly larger in patients with clinically significant prostate cancer compared to those without (7.8 mm vs. 7.3 mm; median 7.0 mm in both groups). Although this difference suggests a trend toward larger lesions being more frequently associated with PCa, the substantial overlap in size distributions indicates that lesion size alone has limited discriminatory power for risk stratification in PI-RADS 3 cases. ROC analysis further confirmed this, identifying 12 mm as the optimal threshold (Youden’s index) with a specificity of 92.8% but a very low sensitivity of 16.6% (AUC 0.52). These results underline that lesion size has poor discriminative ability for predicting csPCa and is not suitable as a standalone stratification criterion. Conversely, the number of prior biopsies (*p* = 0.652) and lesion localization (*p* = 0.587) were not significantly associated with cancer detection.

Regarding csPCa, PSA density again showed the strongest association (*p* < 0.001), with increasing values correlating with higher risk. Age (*p* < 0.001) and PSA level (*p* = 0.028) were also significantly associated. Prostate volume showed an inverse relationship (*p* < 0.001), suggesting a higher csPCa risk in smaller prostates. Unlike all PCa, lesion diameter was not significantly associated with csPCa (*p* = 0.13). However, csPCa was detected more frequently in biopsy-naïve patients (*p* = 0.03) and in posteriorly located lesions (*p* = 0.018). When stratified by lesion location, detection rates were highest in the posterior prostate (39.0%), followed by the anterior transition zone (37.0%) and AFMS (32.3%) (*p* < 0.01). For clinically significant cancers, detection rates were 17.9%, 15.1%, and 12.9%, respectively, with a significant difference between AFMS and posterior lesions (*p* < 0.01).

**Fig. 3:**
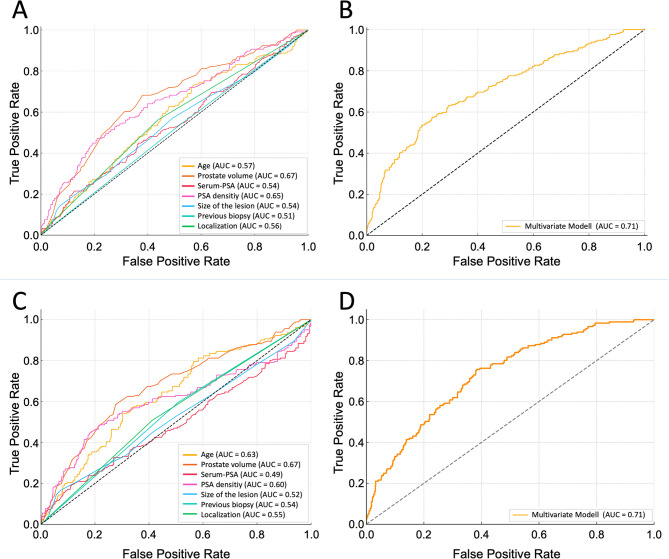
Receiver Operating Characteristic (ROC) curves for univariate and multivariate logistic regression models predicting prostate cancer (all PCA: **A** and **B**) and clinically significant prostate cancer (csPCa: **C** and **D**) in patients with PI-RADS 3 lesions. **A** and **C** Each curve represents the diagnostic performance of a single clinical variable: age, prostate volume, serum PSA, PSA density, localization and lesion size. **B** and **D** Diagnostic performance of the multivariate logistic regression model in distinguishing between patients with and without prostate cancer, with the area under the curve (AUC) quantifying its overall discriminative ability

All variables with significant univariate associations were included in the multivariate analysis, which confirmed their independent predictive value (Tables 3 and 4; Fig. 3).

**Table 3:** Association between overall Prostate Cancer (all PCA) and other clinical factors - Left column: univariate analysis, right column: multivariate analysis

		**Univariable Analysis vs.allPCa** OddsRatio (95% CI), *p*-value	**Multivariable Analysis vs.allPCa** OddsRatio (95% CI), *p*-value	
Age in years		1.03 (1.01–1.05), *p* = 0.003	1.03 (1.01–1.05), *p* = 0.004	
Previous biopsies		0.93 (0.69–1.25), *p* = 0.652	0.79 (0.56–1.1), *p* = 0.16	
Prostate volume in mL		0.97 (0.97–0.98), *p* < 0.001	0.99 (0.98–1.00.98.00), *p* = 0.066	
Serum-PSA in ng/mL		1.98 (1.79–2.18), *p* < 0.001	0.96 (0.90–1.01), *p* = 0.137	
PSA density in ng/mL/cm^3^		25.05 (16.25–33.85), *p* < 0.001	48.89 (21.4–76.38.4.38), *p* = 0.0009	
Localization of the lesion		0.81 (0.61–1.01), *p* = 0.587	0.87 (0.74–1.00.74.00), *p* = 0.74	
Size of the lesion in mm		1.07 (1.05–1.08), *p* = 0.045	1.04 (0.99–1.08), *p* = 0.051

**Table 4:** Association between clinically significant Prostate Cancer (csPCa) and other clinical factors - Left column: univariate analysis, right column: multivariate analysis

	Univariable Analysis vs. csPCa OddsRatio (95% CI), *p*-value	Multivariable Analysis vs.csPCa OddsRatio (95% CI), *p*-value
Age in years	1.06 (1.04–1.09), *p* < 0.001	1.07 (1.04–1.09), *p* < 0.001
Previous biopsies	0.70 (0.51–0.97), *p* = 0.03	0.55 (0.39–0.80), *p* = 0.0015
Prostate volume in mL	0.98 (0.97–0.98), *p* < 0.001	0.98 (0.97–0.99), *p* = 0.0004
Serum-PSA in ng/mL	2.30 (1.84–2.86), *p* = 0.028	1.02 (0.96–1.07), *p* = 0.58
PSA density in ng/mL/cm^3^	22.17 (7.65–64.28), *p* < 0.001	6.71 (0.62–72.37), *p* = 0.117
Localization of the lesion	1.47 (1.07–2.03), *p* = 0.018	1.42 (1.01–1.99), *p* = 0.045
Size of the lesion in mm	1.03 (0.99–1.07), *p* = 0.13	1.03 (0.99–1.08), *p* = 0.12

### Special subgroups

In two subgroups—normal digital rectal examination, PSA < 10 ng/mL, and PSA density < 0.15 ng/mL/cm³, with or without prior biopsy—low overall cancer detection and very low clinically significant prostate cancer (csPCa) rates were observed. Of 230 and 594 biopsies, 87.8% and 88.2% showed no tumor evidence, respectively. Only 3.9% and 4.7% of csPCa would have been missed, without any ISUP grade group 4 or 5 tumors overlooked (Table 5).

**Table 5:** Detection rates of selected subgroups – in parentheses the potentially missed carcinomas

Subgroups of specific clinical variables	Detection Rate – all PCa	Detection Rate – csPCa
Negative DRE Serum PSA < 10 ng/mL PSA-Densitiy < 0.15 ng/mL/cm^3^ Previous Biopsy Negative	12.2% (28/230)	3.9% (9/230) 3x ISUP 3 6x ISUP 2 No ISUP 4 or 5
Negative DRE Serum PSA < 10 ng/mL PSA-Densitiy < 0.15 ng/mL/cm^3^	11.8% (70/594)	4.7% (28/594) 11x ISUP 3 17x ISUP 2 No ISUP 4 or 5

The specificity of the combined clinical parameters was 90% and 86%, indicating high accuracy in correctly identifying patients without csPCa. These findings reflect a high true negative rate and suggest that the combined use of PSA, DRE, and PSA density effectively excludes csPCa, supporting the potential for biopsy deferral in low-risk individuals. Two patient examples are provided in the Supplementary Appendix (Figures S2 and S3).

## Discussion

### Ambiguity of PI-RADS 3 categorization

PI-RADS 3 lesions represent equivocal findings on mpMRI, indicating an uncertain likelihood of clinically significant prostate cancer (csPCa) [7]. Approximately 17% of prostate MRI scans yield PI-RADS 3 findings, yet only 15–20% of these harbor csPCa [14, 16, 24]. In contrast, detection rates are significantly higher for PI-RADS 4 (40–50%) and PI-RADS 5 (70–90%) [25, 26]. The PI-RADS 3 score reflects imaging features that suggest malignancy but do not fulfill higher category criteria across T2-weighted, diffusion-weighted (DWI), and dynamic contrast-enhanced (DCE) sequences. This indeterminate score creates a clinical challenge, as it neither confirms nor excludes csPCa with confidence [7].

In our cohort, we observed PCa in 36.9% of cases, with csPCa in 15.8%. Higher detection rates were associated with greater lesion size, elevated PSA, posterior lesion location, smaller prostate volume, and higher PSA density—findings consistent with the literature. Our regression models demonstrated consistent and clinically meaningful associations. The wide confidence intervals observed for PSA density are likely attributable to the limited number of csPCa events and the intrinsic collinearity with PSA and prostate volume. Although the Hosmer–Lemeshow test suggested minor miscalibration, the low Brier score (0.078) and the overall calibration plot indicate that the models achieved acceptable predictive accuracy. These results support the robustness of our findings and highlight the applicability of the models for risk estimation in this cohort. Moreover, we identified subgroups with minimal csPCa risk, where biopsy avoidance appears justified.

### Likelyhood of clinically significant cancer in PI-RADS 3 lesions

Despite their intermediate classification, PI-RADS 3 lesions occasionally harbor csPCa. Meta-analyses of PI-RADS v2.0/v2.1 report csPCa detection rates between 13 and 20% [14, 16]. Our finding of 16.2% integrates coherently into the overall context. In contrast, csPCa is identified in up to 50% of PI-RADS 4 and 69% of PI-RADS 5 lesions [5, 6, 10, 11, 19]. This underscores the comparatively low malignancy potential associated with PI-RADS 3 lesions. Detection rates vary by cohort characteristics and imaging protocols. Some single-center studies report higher csPCa rates—up to 37.5%—suggesting improved MRI performance or patient selection [27]. However, such elevated rates are exceptions rather than the norm. A comprehensive review documented biopsy positivity ranging from 0 to 25%, reflecting broad heterogeneity [27]. Collectively, the consensus is that only one in five – or fewer – PI-RADS 3 lesions are csPCa, while most are benign or low-grade Gleason 3 + 3 tumors, highlighting the diagnostic challenge and therapeutic implications associated with this intermediate-risk category.

In this context, quantitative approaches such as apparent diffusion coefficient (ADC) analysis may provide additional diagnostic value. Pepe et al. demonstrated that an ADC threshold of 0.747 × 10⁻³ mm²/s achieved higher diagnostic accuracy (84%) and a superior AUC (0.81) compared to PI-RADS ≥ 3 (63.6%, AUC 0.71) for detecting Gleason ≥ 7 tumors [28]. These findings indicate that ADC evaluation could support clinicians in decision-making for patients with PI-RADS < 3 who remain at risk for clinically significant prostate cancer. In our cohort, the detection rates of clinically significant prostate cancer (csPCa) were indeed comparable between biopsy-naïve men and those undergoing repeat biopsy. This can be explained by the mpMRI-based preselection of all patients, which enriched both groups with a higher pre-test probability of harboring csPCa. Moreover, men referred for repeat biopsy typically presented with persistent clinical suspicion, resulting in a risk profile similar to biopsy-naïve patients. Importantly, anterior tumors—which historically were often only detected during repeat biopsy—are now routinely identified and targeted at initial biopsy owing to mpMRI guidance. This methodological advance has reduced the historical gap between initial and repeat biopsy cohorts and likely accounts for the comparable detection rates observed in our study.

For the sake of completeness, it should be noted that both transrectal and transperineal MRI-targeted prostate biopsies demonstrate comparable overall detection rates for csPCa, with the transperineal approach offering advantages for anterior tumors. Schieda et al. demonstrated that when image quality is high and MRI lesions are clearly identifiable, targeted biopsy alone is often sufficient, thereby avoiding unnecessary systematic cores, reducing patient risk, and lowering costs. Moreover, the transperineal approach provides additional benefits, particularly for anteriorly located lesions [29, 30].

Accordingly, the current EAU Guidelines recommend the transperineal route as the preferred method due to its lower risk of infectious complications and its favorable impact on antibiotic stewardship [1].

### Limitations of imaging and interobserver variability

The clinical challenge of PI-RADS 3 is compounded by limitations in MRI interpretation and observer variability:

A key challenge in the interpretation of PI-RADS 3 lesions lies in their frequent overlap with benign prostatic conditions.

A major result in our analysis is the inverse correlation between prostate volume and cancer detection: larger prostates are statistically associated with lower detection rates of clinically significant prostate cancer (csPCa). This is likely due to benign conditions, such as chronic prostatitis or post-inflammatory changes in the peripheral zone, which frequently mimic malignant features on MRI—e.g., diffusion restriction or enhancement—without representing true neoplasia [31].

In the transition zone, benign prostatic hyperplasia (BPH) nodules can exhibit atypical imaging characteristics (e.g., heterogeneous T2 signal, restricted diffusion in infarcted nodules) that contribute to equivocal scoring. These benign mimics lower the positive predictive value of PI-RADS 3 and account for a substantial proportion of false-positive findings [31]. In one study, the incidence of PI-RADS 3 findings was significantly elevated in patients with imaging signs of prostatitis, yet the csPCa detection rate was only 7.7% [31].

PI-RADS 3, by definition, lacks definitive imaging hallmarks of malignancy. Lesions are typically inconspicuous, with only subtle abnormalities on diffusion-weighted imaging or T2-weighted sequences. Early or small tumors may share imaging characteristics with normal anatomical variations. Consequently, image quality, MRI parameters, and reader expertise critically influence lesion visibility and scoring. Even minor technical limitations—motion artifacts, suboptimal resolution—can obscure lesions, increasing the risk of misclassification [25].

Interobserver variability remains a central limitation. Radiologists often disagree on whether a lesion should be scored as PI-RADS 2, 3, or 4. Reported inter-reader agreement is only fair (κ ~ 0.41 for PI-RADS 3–5; κ ~ 0.51 for PI-RADS 4–5), reflecting both ambiguous criteria and varying levels of reader experience [28]. These discrepancies are further compounded by marked variability in image quality across centers, as highlighted by Giganti et al., who demonstrated that differences in scanner field strength (1.5 T vs. 3 T), coil configurations, and sequence parameters such as resolution, b-values, and contrast-enhanced imaging substantially affect prostate MRI quality [32]. Moreover, adherence to PI-RADS v2.1 technical standards is often incomplete in clinical practice, and practical issues such as motion artifacts or suboptimal patient preparation further deteriorate image quality. Together, these factors exacerbate the uncertainty embedded in the “equivocal” PI-RADS 3 category. Importantly, Pepe et al. reported that affiliated centers classified a substantially higher proportion of patients as PI-RADS 3 compared to reference institutions, yet with lower csPCa detection rates (16.6% vs. 26.7%). On expert review, many of these lesions were downgraded or upgraded, underscoring the decisive impact of radiological expertise on diagnostic accuracy [33]. While recent updates in PI-RADS v2.1 have refined criteria, the core diagnostic limitations remain.

Dwivedi et al. demonstrated that advanced mpMRI techniques such as VERDICT-MRI, luminal water imaging, MR fingerprinting, and radiomics can help overcome the limitations of PSA-based screening and TRUS biopsy. Although still under validation, these methods show promise for improving risk stratification and the management of equivocal PI-RADS 3 lesions [34, 35].

Emerging technologies such as radiomics and machine learning hold promise for improved lesion characterization but are not yet standard in clinical practice [21].

### Impact on clinical decision-making

The indeterminate nature of PI-RADS 3 directly translates into a clinical management dilemma: should a PI-RADS 3 lesion prompt an immediate biopsy, or can the patient be safely observed?

This question is challenging because of the trade-offs involved:

The clinical management of PI-RADS 3 lesions is challenging due to the balance between avoiding unnecessary interventions and not missing csPCa. Biopsying all PI-RADS 3 lesions would expose many patients to invasive procedures despite a high rate of non-significant findings—over 80% of these lesions may not yield csPCa [21]. Biopsies carry risks such as bleeding, infection, and sepsis [21, 25] increase healthcare costs, and cause anxiety. Detecting insignificant cancer (Gleason 3 + 3) may lead to overtreatment. Thus, routinely biopsying all PI-RADS 3 lesions risks over-diagnosis and patient harm and should be avoided when possible. On the other hand, deferring biopsy carries a measurable, though relatively low, risk of missing csPCa. Approximately 10–20% of PI-RADS 3 lesions do contain clinically significant cancer [17, 18], and delayed diagnosis can be serious—especially in patients with additional risk factors such as rising PSA or family history. Clinicians must weigh the harms of missed cancer against those of unnecessary procedures, making management complex and controversial. Recent evidence supports a more individualized approach. Risk stratification using clinical and biochemical markers is increasingly used to guide biopsy decisions [20, 22]. Among these, PSA density (PSAD) has proven particularly valuable. A PSAD < 0.15 ng/mL/cc indicates low risk for csPCa, while higher values suggest greater risk. A meta-analysis showed that this threshold safely reduces unnecessary biopsies while maintaining high sensitivity (95–97%) [19]. In line with this, Barrett et al. highlight that the clinical value of mpMRI depends not only on technical standards, but also on consistent patient preparation, structured reporting, and multidisciplinary collaboration. They recommend the systematic use of quality assessment tools, regular audits, and feedback mechanisms to ensure reproducibility and maximize patient benefit [36].

Based on this background, we identified subgroups in which the detection rates are expected to be extremely low, meaning that the vast majority of biopsies in these patients would likely be unnecessary. Variables included in our decision-making process—assuming an unsuspicious digital rectal examination—are the total PSA level, PSA density, and, optionally, a history of prior biopsy. Using this approach, only 3.9% and 4.7% of clinically significant carcinomas would be missed, none of which would be classified as ISUP grade 4 or 5.

Many guidelines now support observation for PI-RADS 3 with low PSAD [1, 2, 37, 38]. Mjaess et al. showed that PSA density was the only significant predictor among 790 PI-RADS 3 cases, and that a threshold of < 0.13, especially < 0.09, allows safe avoidance of biopsy [37]. These findings have been validated in multiple studies [15, 16, 22, 38]. In line with this, Roscigno et al. further demonstrated that a PSAD ≥ 0.20 ng/mL² significantly increased the risk of disease reclassification in active surveillance cohorts, particularly in patients with PI-RADS 4–5 lesions, whereas low PSAD values (< 0.10 ng/mL²) were associated with a more favorable prognosis [39]. These results underscore the complementary role of PSAD in refining mpMRI-based risk stratification and highlight its potential to safely reduce unnecessary biopsies, especially in men with equivocal PI-RADS 3 lesions. By standardising technical requirements and providing a simplified scoring system, PI-QUAL v2 enhances the reliability of prostate MRI, thereby improving diagnostic confidence and reducing unnecessary repeat examinations, ultimately supporting more efficient and patient-centred care. A key question for future research is whether such technical advances can translate into a measurable improvement in the management and outcomes of patients with equivocal PI-RADS 3 lesions [40].

Recent evidence also suggests that 68Ga-PSMA PET/CT may contribute to biopsy reduction by providing a higher negative predictive value compared to mpMRI, although its detection rate for csPCa is not superior to systematic transperineal saturation biopsy [41]. Nevertheless, the high costs and limited availability of PSMA PET/CT currently restrict its routine implementation in clinical practice.

Additional clinical variables—age, PSA kinetics, family history, race—and novel biomarkers (e.g., 4Kscore, PHI, PCA3) can further refine risk. Such tools are useful in borderline cases to guide management [1, 2, 26]. Another approach is short-term monitoring. Instead of immediate biopsy, some protocols recommend repeat MRI after 6–12 months. If the lesion remains stable or regresses, biopsy may be avoided; progression may prompt it. While not standardized, this strategy is commonly applied in select patients. One study showed that 55% of PI-RADS 3 lesions remained stable over ~ 2 years, with only a few progressing to csPCa [20]. This supports surveillance as a viable option, similar to strategies used in low-risk prostate cancer.

In summary, diagnosing csPCa in PI-RADS 3 is not a binary decision. Management relies on integrating MRI findings with the patient’s overall risk profile. Shared decision-making is essential—patient values, preferences, and clinical judgment must all play a role in whether to biopsy or monitor.

## Conclusion

PI-RADS 3 represents an intermediate risk category – most such lesions do not contain aggressive cancer, yet a meaningful minority does. This uncertainty is magnified by limitations of imaging (overlap with benign conditions and reader variability) that make it difficult to confidently distinguish which PI-RADS 3 lesions are dangerous. As a result, management must be carefully individualized. Clinicians employ adjunctive measures like PSA density, clinical risk factors, and sometimes repeat imaging to stratify the likelihood of cancer. The decision of whether to proceed with biopsy or to monitor must balance the risk of missing a significant cancer against the downsides of unnecessary biopsy. In essence, the PI-RADS 3 dilemma exemplifies the broader challenge in prostate cancer early detection: how to avoid both overdiagnosis and underdiagnosis. Ongoing research – including advanced imaging techniques, biomarkers and the use of artificial intelligence – is aimed at improving risk discrimination in this equivocal group.

Until then, the management of PI-RADS 3 will remain a clinical gray zone requiring expert judgment, patient counseling, and often a tailored combination of surveillance and targeted intervention.

## Supplementary Information

Supplementary Material 1: Table S1. Inclusion and exclusion criteria

Supplementary Material 2: Table S2. Number of prior biopsies

Supplementary Material 3: Figure S1. CONSORT-Style flow diagram illustrating patient selection, patients excluded, those fulfilling the inclusion and exclusion criteria and detection rates per patient and per lesion

Supplementary Material 4: Figure S2. Representative example of a potential clinical workflow leading to surveillance

Supplementary Material 5: Figure S3. Representative example of a potential clinical workflow leading to fusion biopsy

## Data Availability

The datasets generated and analyzed during the current study are subject to a six-month embargo period. After this time, they will be available upon reasonable request from the corresponding author.

## Abbreviations

AFMS: AFMS
aTZ: Anterior transition zone
CI: Confidence interval
cs: Clinically significant
DRE: Digital rectal examination
ISUP: International Society of Urological Pathology
(mp)MRI: (Multiparametric) magnetic resonance imaging
MVA: Multivariable analysis
non-cs: Clinically non-significant
OR: Odds ratio
PCa: Prostate cancer
PI-RADS: Prostate imaging reporting and data system
PSA: Prostate specific antigen
TRUS: Transrectal ultrasound
US: Ultrasound
UVA: Univariable analysis
