# Supplementary material for: To biopsy or not biopsy, that is the question - PI-RADS 3 prostate lesions – validation of clinical and radiological parameters for biopsy decision-making
Source: BMC Urol. 2025 Nov 1;25:274. doi: 10.1186/s12894-025-01986-2 (PMC12579397; doi:10.1186/s12894-025-01986-2)
Supplement: Supplementary file 1 — Supplementary Material 1: Table S1. Inclusion and exclusion criteria [file 12894_2025_1986_MOESM1_ESM.docx]

| **Table S1**: Inclusion and exclusion criteria |
| --- |
| **Inclusion criteria (all must be fulfilled):** |
| - Male patients aged ≥ 18 years undergoing clinical evaluation for suspected prostate cancer - Availability of pre-biopsy serum PSA measurement within 3 months before mpMRI - Elevated PSA (age-specific) OR abnormal PSA kinetics (e.g., PSA velocity > 0,75 ng/ml/year) - Negative digital rectal examination (DRE) - mpMRI performed prior to biopsy according to PI-RADS version 2.1 standards - At least one mpMRI lesion scored PI-RADS 3 - Eligibility and consent for MRI–ultrasound fusion-guided biopsy - Written informed consent to participate in the study and for data analysis. |
| **Exclusion criteria (excluded if *any* of the following criteria applied):** |
| - mpMRI with PIRADS 4 oder 5 - Prior definitive prostate treatment (e.g., radical prostatectomy, external beam radiotherapy, brachytherapy, cryoablation, HIFU, focal laser ablation). - Ongoing androgen deprivation therapy or prior systemic therapy for prostate disease. - Incomplete clinical or imaging data (e.g., missing PSA, missing mpMRI sequences). - Contraindications to MRI (e.g., non-MRI-compatible implants, severe claustrophobia). - Contraindications to gadolinium-based contrast agents (e.g., severe renal insufficiency, prior allergic reaction). - Severe comorbidities precluding biopsy under local or general anesthesia. - Inability to provide written informed consent. |
