# Supplementary material for: To biopsy or not biopsy, that is the question - PI-RADS 3 prostate lesions – validation of clinical and radiological parameters for biopsy decision-making
Source: BMC Urol. 2025 Nov 1;25:274. doi: 10.1186/s12894-025-01986-2 (PMC12579397; doi:10.1186/s12894-025-01986-2)
Supplement: Supplementary file 2 — Supplementary Material 2: Table S2. Number of prior biopsies [file 12894_2025_1986_MOESM2_ESM.docx]

| **Table S2**: number of prior biopsies | |
| --- | --- |
| **Number of prior biopsies** | **N (%) of patients** |
| 0 (biopsy-naïve) | 351 (52.3 %) |
| 1 | 198 (29.5 %) |
| 2 | 65 (9.7 %) |
| 3 | 32 (4.8 %) |
| 4 | 14 (2.1 %) |
| 5 | 9 (1.3 %) |
| 6 | 1 (0.1 %) |
| 7 | 1 (0.1 %) |
|  | 671 (100 %) |
