# Supplementary figures and images for: To biopsy or not biopsy, that is the question - PI-RADS 3 prostate lesions – validation of clinical and radiological parameters for biopsy decision-making
Source: BMC Urol. 2025 Nov 1;25:274. doi: 10.1186/s12894-025-01986-2 (PMC12579397; doi:10.1186/s12894-025-01986-2)

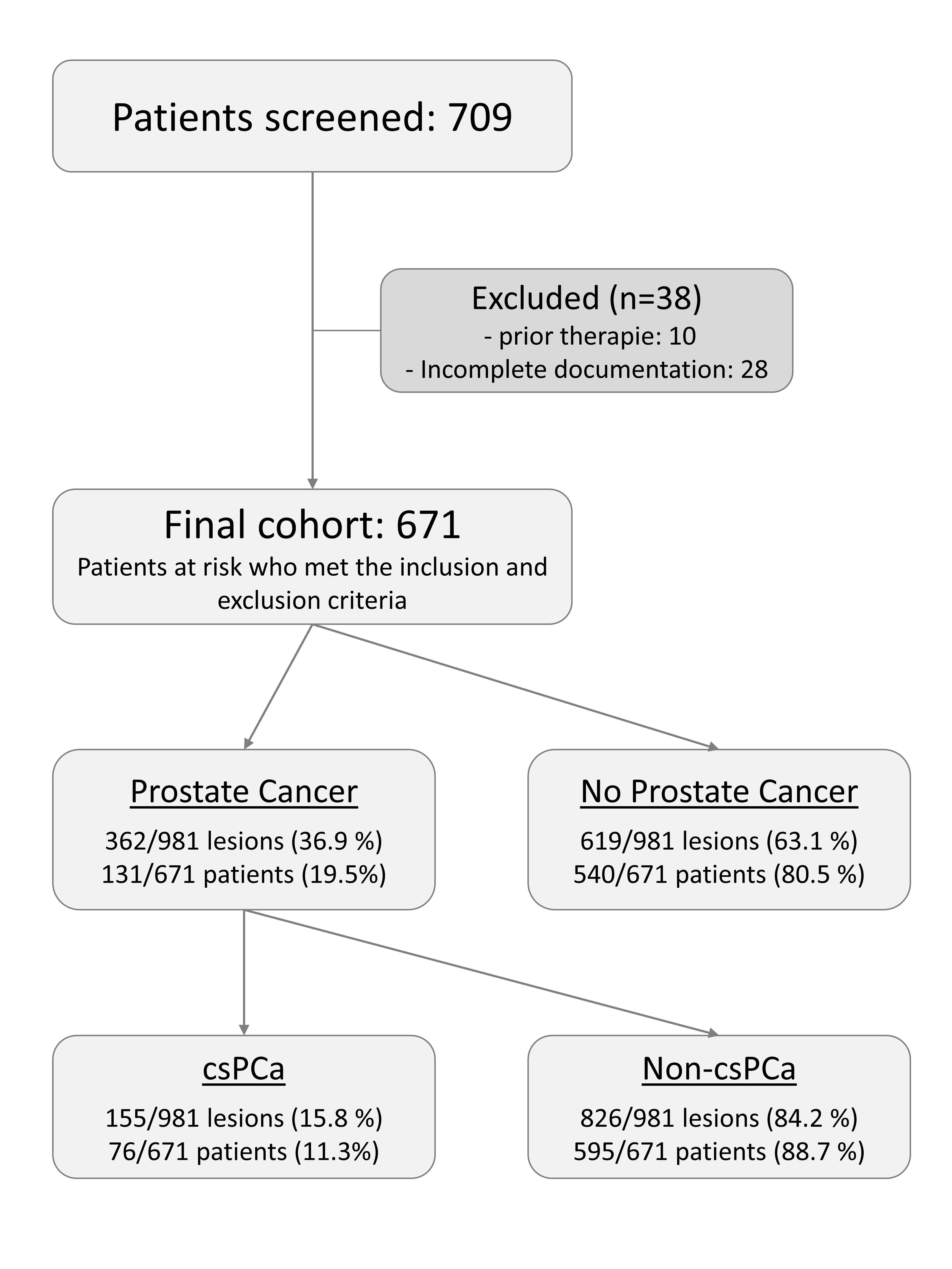

Supplement: Supplementary file 3 — Supplementary Material 3: Figure S1. CONSORT-Style flow diagram illustrating patient selection, patients excluded, those fulfilling the inclusion and exclusion criteria and detection rates per patient and per lesion [file 12894_2025_1986_MOESM3_ESM.jpg]

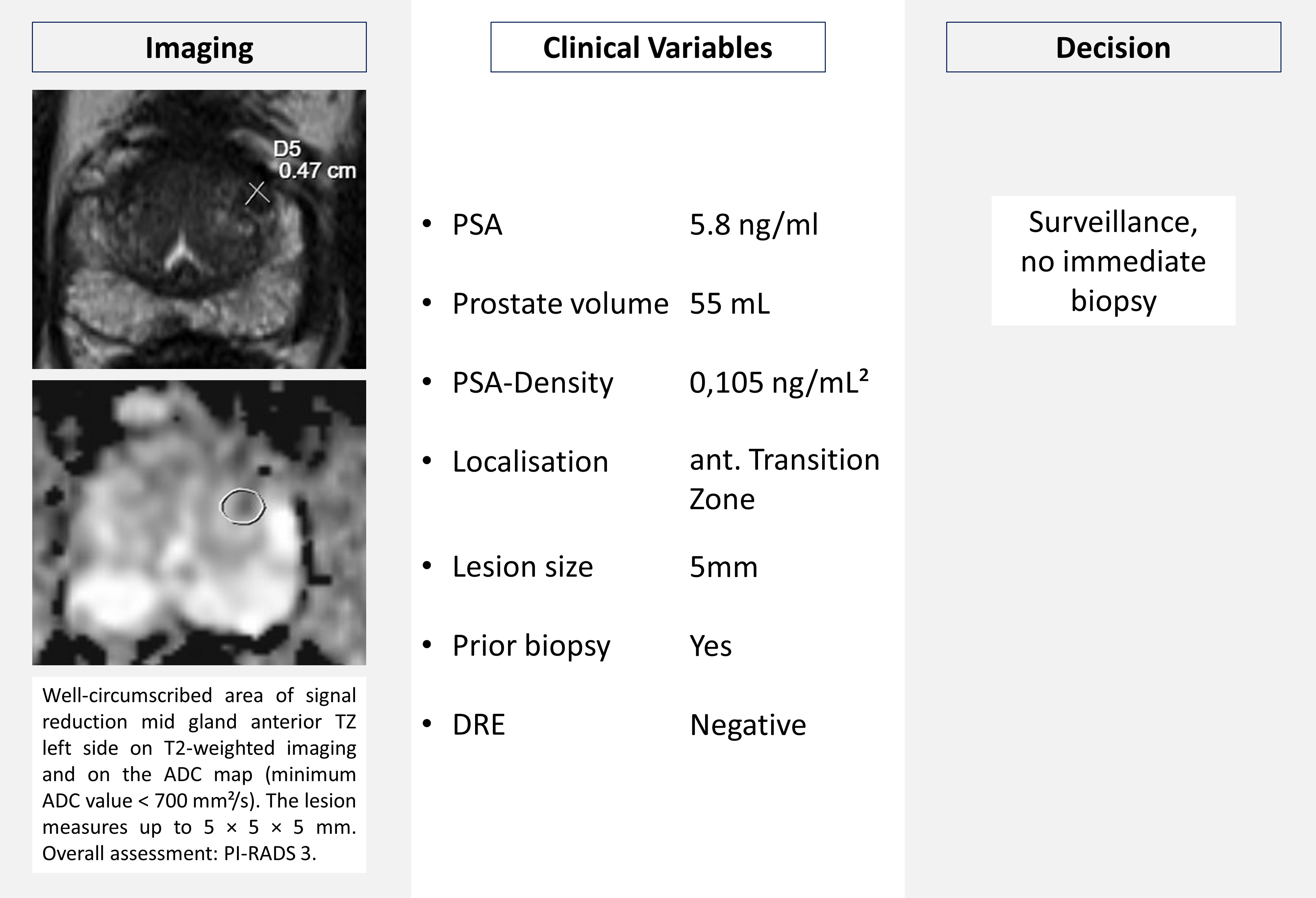

Supplement: Supplementary file 4 — Supplementary Material 4: Figure S2. Representative example of a potential clinical workflow leading to surveillance [file 12894_2025_1986_MOESM4_ESM.jpg]

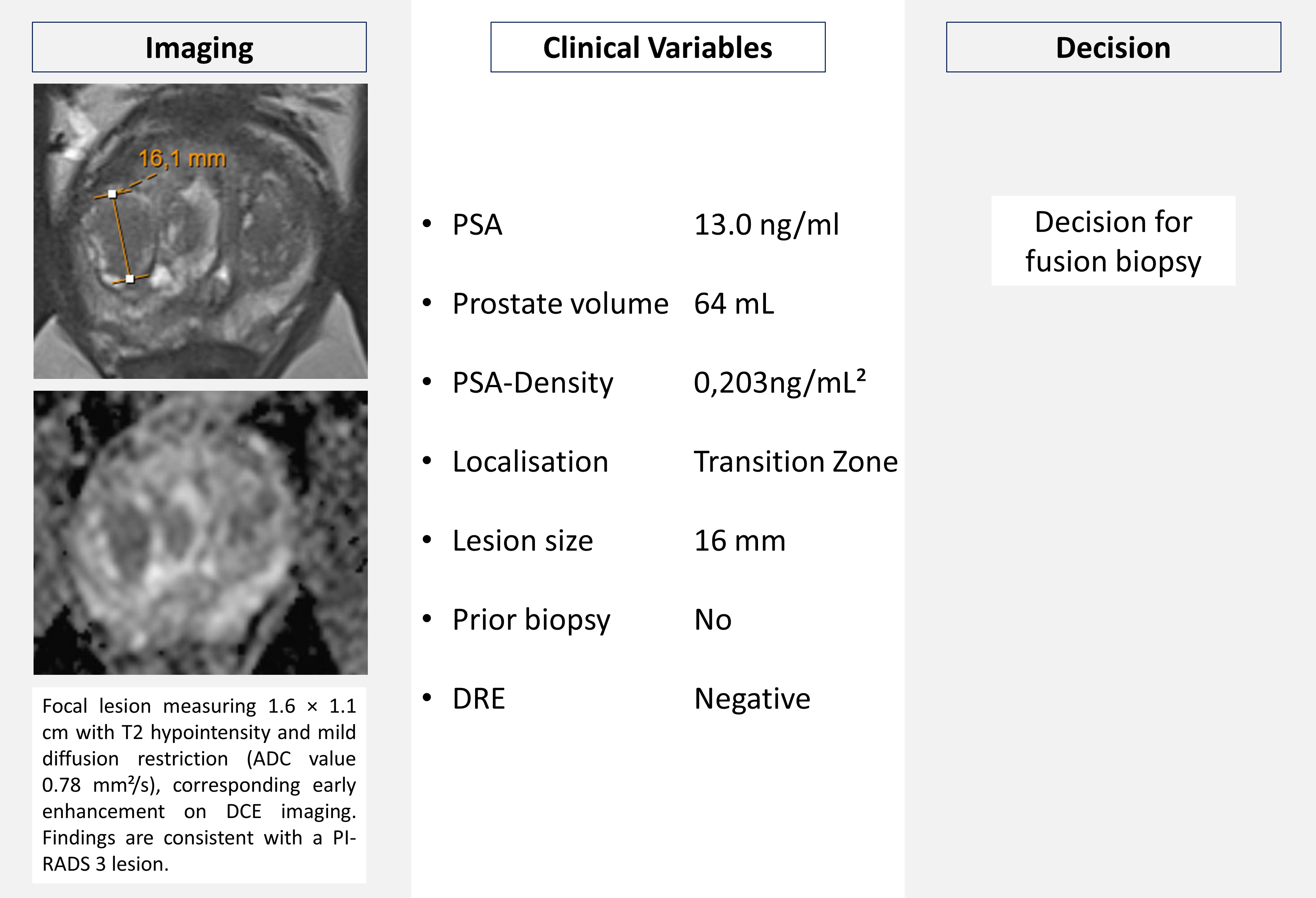

Supplement: Supplementary file 5 — Supplementary Material 5: Figure S3. Representative example of a potential clinical workflow leading to fusion biopsy [file 12894_2025_1986_MOESM5_ESM.jpg]
